# Comment on: “Risk Factors of Thrombocytopenia After Cardiac Surgery with Cardiopulmonary Bypass”

**DOI:** 10.21470/1678-9741-2023-0010

**Published:** 2023-07-18

**Authors:** Cihan Bedel, Fatih Selvi, Günay Yıldız, Ayşe Şeyma Uysal

**Affiliations:** 1 Department of Emergency Medicine, Health Science University, Antalya Training and Research Hospital, Antalya, Turkey. E-mail: cihanbedel@hotmail.com

We have read the article by Yan et al.^[1]^ entitled “Risk Factors of Thrombocytopenia After Cardiac Surgery with Cardiopulmonary Bypass” with great interest. The authors emphasized that thrombocytopenia is common after cardiac surgery, and the risk factors for postoperative thrombocytopenia include age, body surface area, preoperative thrombocytopenia, and duration of cardiopulmonary bypass. First, we congratulate the authors for their valuable contributions to the literature. However, we would like to highlight some issues that may require further attention.

Heparin is a widely used drug in clinical practice, and one of the side effects associated with this drug is thrombocytopenia. Considering all patients using heparin, the risk and rate of development of thrombocytopenia vary regardless of the type of use or the dose used^[2]^. Some other drugs may also cause immune thrombocytopenia, *e.g.*, anticonvulsive drugs, coumadin, beta-lactam group antibiotics, oral antidiabetics, diuretics, nonsteroidal anti-inflammatory drugs, sulfonamides, and antituberculosis drugs^[3,4]^. It would be more appropriate for the authors to consider the conditions that may cause thrombocytopenia, such as these drugs.

Thrombocytopenia, which is an important cause of mortality in patients hospitalized in the intensive care unit, especially in the postoperative period, may occur due to many etiological factors. Infections that may develop in postoperative patients, especially cases of bacteremia, and disseminated intravascular coagulation that may develop due to infection or multiorgan dysfunction may be included in the etiology of thrombocytopenia^[5]^. We think that it would be appropriate for the authors to mention the postoperative infection situation that may develop during hospitalization.
